# Fibrin Clot Strength in Patients with Diabetes Mellitus Measured by Thrombelastography

**DOI:** 10.1155/2018/4543065

**Published:** 2018-02-04

**Authors:** Benjamin T. Maatman, Glen Schmeisser, Rolf P. Kreutz

**Affiliations:** Krannert Institute of Cardiology, Indiana University School of Medicine, Indianapolis, IN, USA

## Abstract

**Background:**

Patients with diabetes mellitus (DM) exhibit increased risk of recurrent myocardial infarction. Maximal clot strength measured by thrombelastography (TEG) is a risk factor for recurrent ischemic events. We hypothesized that diabetic subjects exhibit increased fibrin clot strength in platelet-poor plasma and that glycemic control correlates with maximal fibrin clot strength.

**Methods:**

We collected plasma samples from subjects with known or suspected coronary artery disease undergoing cardiac catheterization (*n* = 354). We measured kaolin-activated TEG in platelet-poor citrate plasma. Time to fibrin formation (R), clot formation time (K), and maximal fibrin clot strength (MA) were recorded.

**Results:**

Plasma fibrin MA was increased among subjects with DM (*n* = 152) as compared to non-DM (*n* = 202) (37.0 ± 8 versus 34.1 ± 8 mm; *p* < 0.001). Hemoglobin A1c (HbA1c) (*ρ* = 0.22; *p* = 0.001) and fibrinogen (*ρ* = 0.29; *p* < 0.001) correlated with fibrin MA. In multivariable regression analysis, DM remained significantly associated with plasma MA after adjustment for fibrinogen level (*p* = 0.003).

**Conclusions:**

Subjects with diabetes mellitus exhibit increased maximal fibrin clot strength measured by TEG in platelet-poor plasma.

## 1. Introduction

Diabetes mellitus (DM) leads to increased risk of coronary artery disease (CAD) and is considered a CAD risk equivalent [1–5]. Hyperglycemia during acute coronary syndrome (ACS) has been associated with worse outcomes even in the absence of overt diabetes [6]. DM is associated with elevated fibrinogen, increased thromboxane A2, reduced platelet nitric oxide synthesis, as well as increased plasminogen activator inhibitor-1 (PAI-1) release from platelets leading to inhibition of thrombolysis [7–11].

Thrombelastography (TEG) is an established clinical laboratory method for analysis of coagulation and fibrinolysis and allows for quantitative measurement of the dynamic processes of clot formation and clot strength [12, 13]. We have previously demonstrated the feasibility of measurement of plasma fibrin clot strength by TEG in platelet-poor plasma and correlation with whole blood fibrin TEG measurements [14]. Elevated whole blood as well as platelet-poor plasma maximal clot strength by TEG (TEG-MA) has been associated with an increased risk of ischemic events after coronary stenting [15–17]. Subjects with high plasma clot strength showed a 3.8-fold increased risk for cardiovascular death or myocardial infarction after percutaneous coronary intervention [17].

We hypothesized that subjects with DM exhibit increased fibrin clot strength (TEG-MA) in platelet-poor plasma as compared with controls and that extent of glycemic control correlates with maximal fibrin clot strength.

## 2. Materials and Methods

### 2.1. Patients

The study protocol was approved by the Indiana University Institutional Review Board for Research. All enrolled individuals gave written informed consent. Subjects with known or suspected coronary artery disease who were referred for cardiac catheterization were included in this study. Blood samples were obtained prior to cardiac catheterization or hospital discharge. Plasma samples were stored as part of a biobank registry in patients with cardiovascular disease (Genetics and Cardiac Catheterization (GENCATH) study).

### 2.2. Blood Samples

Whole blood was collected by peripheral venipuncture in 3.2% citrate after discarding of initial blood draw. We obtained platelet-poor plasma (PPP) by centrifugation at 2000 ×g for 15 min. Plasma was stored at −80°C and thawed immediately before analysis.

### 2.3. Subjects with Diabetes Mellitus

Hemoglobin A1c (HbA1c) was recorded if there was a value present during chart review within three months of the blood sample collection. Within the insulin-dependent subpopulation of the diabetic group, the total daily administered insulin dose was recorded and this was defined as the sum of short-acting plus long-acting insulin as obtained from the patient's medication list.

### 2.4. Thrombelastography (TEG)

Kaolin-activated TEG (Haemonetics, USA) was performed in all subjects (*n* = 354) using platelet-poor citrate plasma samples according to the manufacturer's instructions. Citrate plasma was mixed with kaolin and then loaded in a heparinase-coated cup containing 20 ml of CaCl2 [18]. Thrombelastography was stopped after time to fibrin formation (R), clot formation time (K), and maximal clot strength (MA) were recorded. In a subset of subjects (*n* = 74), kaolin-activated TEG was also performed in the same fashion using whole blood citrate samples.

### 2.5. Enzyme-Linked Immuno Assays (ELISA)

Fibrinogen (Fibrinogen (human) ELISA kit; Alpco Diagnostics, Salem, New Hampshire, USA), Plasminogen Activator Inhibitor-1 (PAI-1 (human) ELISA kit; Raybiotech, Norcross, Georgia, USA) and factor XIIIa (Zymutest Factor XIII-A, Aniara, West Chester, Ohio, USA) were measured by ELISA in citrate platelet-poor plasma according to the manufacturer's instructions.

### 2.6. Statistical Analysis

We conducted statistical analysis with SPSS 24 (IBM, USA). We defined statistical significance as a *p* value less than 0.05, with all tests being conducted two sided. All the values are mean ± SD. We used unpaired, two-sided Student's *t*-test to compare normally distributed continuous data between two groups after assessing normal distribution of continuous data by the Kolmogorov-Smirnov test. We used the χ^2^ test to compare categorical variables. To calculate correlation between thrombelastography parameters (MA, R, and K) and other continuous variables, Spearman's rho was used. Multivariable linear regression analysis was performed with TEG-MA as the dependent variable and diabetes mellitus as the covariate with forward stepwise adjustment for remaining clinical variables and fibrinogen level.

## 3. Results

We enrolled 152 diabetic and 202 nondiabetic subjects (total *n* = 354). Baseline characteristics of the study subjects are displayed in Table 1. Diabetic subjects had higher body mass index (BMI) and higher prevalence of hypertension and hyperlipidemia but lower prevalence of smoking (Table 1). HbA1C was higher in the diabetic group as compared to nondiabetic subjects (7.92 ± 0.5% versus 5.82 ± 1.8%; *p* < 0.001) (Table 1). Among the insulin-dependent diabetic subjects, the average total daily dose of insulin was 93 units (Table 1).

**Figure 1:**
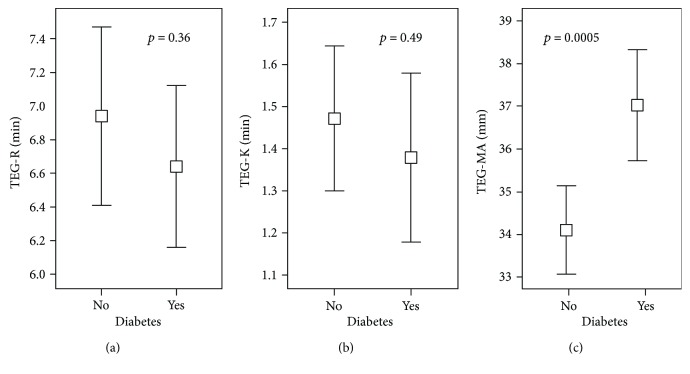
Thrombelastography measures between subjects with diabetes mellitus and without diabetes mellitus in platelet-poor plasma. (a) TEG-R: time to fibrin formation. (b) TEG-K: clot formation time. (c) TEG-MA: maximal fibrin clot strength. Mean ± 95% confidence interval.

**Figure 2:**
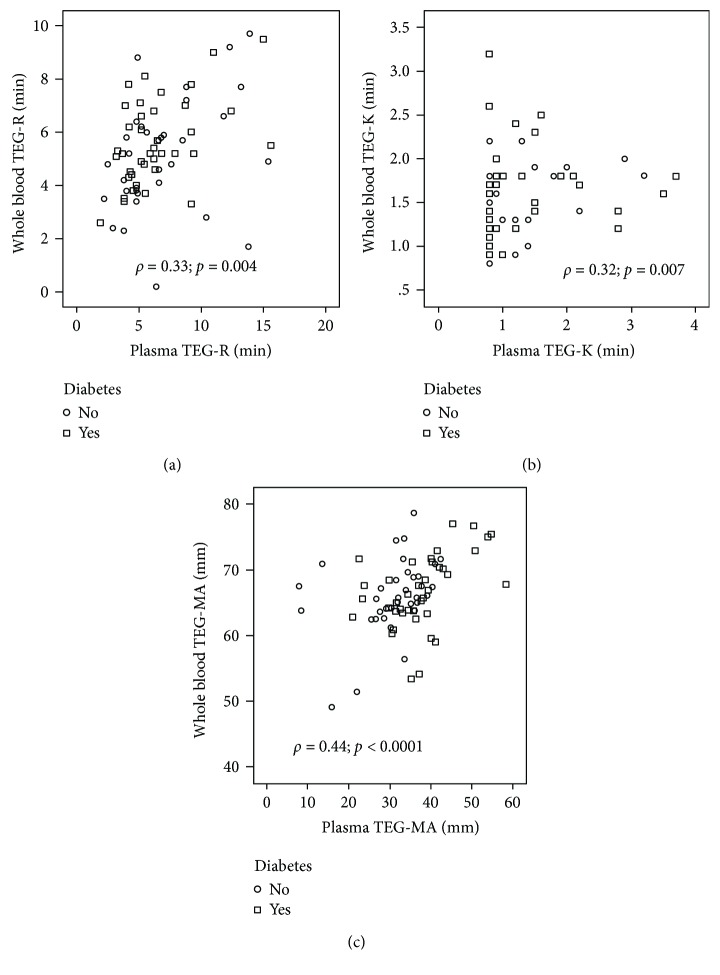
Correlation between whole blood and platelet-poor plasma TEG measurements in a subgroup of the overall study population (*n* = 74) divided between subjects with and without diabetes mellitus. (a) TEG-R: time to fibrin formation. (b) TEG-K: clot formation time. (c) TEG-MA: maximal clot strength.

Maximal fibrin clot strength in platelet-poor plasma (plasma TEG-MA) was higher among subjects with diabetes as compared to nondiabetics (37.0 ± 8 versus 34.1 ± 8 mm; *p* < 0.001) (Table 2, Figure 1). There was no significant difference in time to initial fibrin formation (plasma TEG-R) in the diabetic population (6.6 ± 3 versus 6.9 ± 3.8 min; *p* = 0.36) or clot formation time (plasma TEG-K) (1.38 ± 1.3 versus 1.47 ± 1.2 min; *p* = 0.49) (Figure 1, Table 2). Among the subgroup of individuals (*n* = 74) who had both plasma and whole blood TEG assays completed, there was no significant difference in whole blood TEG measurements between the DM and non-DM groups (Table 2). Whole blood and platelet-poor plasma TEG measurements correlated for all measures but most strongly for TEG-MA (*ρ* = 0.44; *p* < 0.0001) (Figure 2).

There was a nonsignificant trend towards shorter clot formation time (plasma TEG-K: 1.12 ± 0.6 versus 1.5 ± 1.4 min; *p* = 0.08) in insulin-dependent diabetics compared to non-insulin-dependent diabetics. There was no significant difference in plasma TEG-MA (38.1 ± 9 versus 36.5 ± 8 mm; *p* = 0.27) and time to fibrin formation between insulin-dependent and non-insulin-dependent diabetics (plasma TEG-R: 6.3 ± 2.3 versus 6.8 ± 3.3 min; *p* = 0.37) (Table 3). The differences in plasma TEG-MA remained unchanged between DM and non-DM subjects after exclusion of subjects with HbA1C in the prediabetic range (5.7–6.4% as defined by American Diabetes Association [19]) (plasma TEG-MA: 37.3 ± 8 versus 34.3 ± 7 mm; *p* < 0.001).

**Figure 3:**
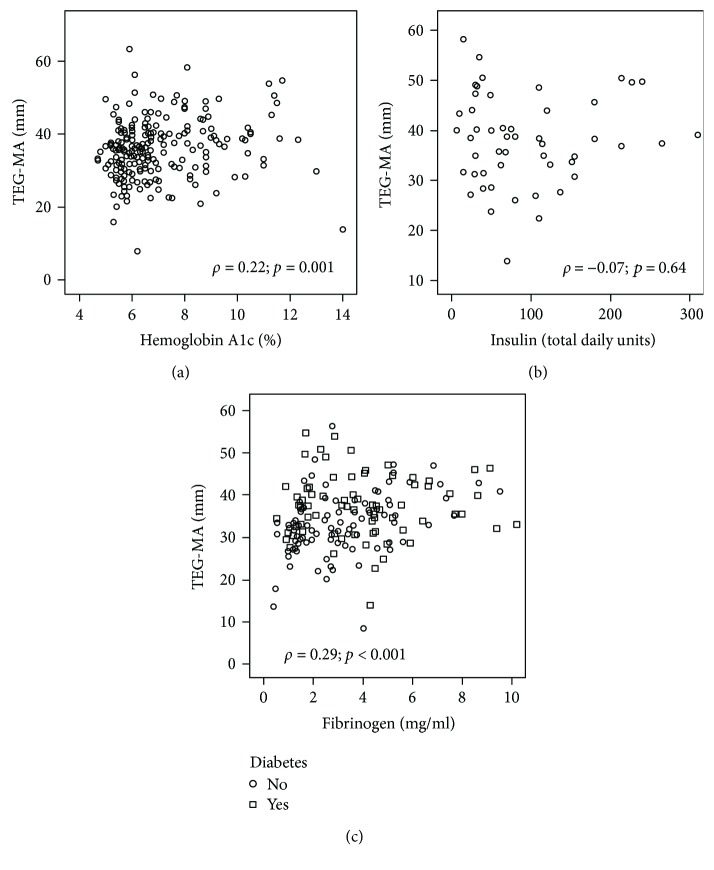
Scatterplot demonstrating maximal plasma fibrin clot strength (TEG-MA), hemoglobin A1c (a), insulin total daily dose (units) (b), and fibrinogen (c).

HbA1c correlated with plasma TEG-MA (*ρ* = 0.22, *p* = 0.001), with higher TEG-MA in subjects with elevated HbA1C and, hence, poorer control of their DM (Figure 3). There was no significant correlation between daily insulin dose and TEG-MA among patients with insulin-dependent DM (*ρ* = −0.07; *p* = 0.64) (Figure 3).

Fibrinogen was elevated in subjects with diabetes mellitus (4.3 ± 4 versus 3.2 ± 2 mg/ml, *p* = 0.027) (Table 1). Fibrinogen correlated with plasma fibrin TEG-MA (*ρ* = 0.29; *p* < 0.001; Figure 3) but not whole blood TEG-MA (*ρ* = 0.14; *p* = 0.45). PAI-1 and factor XIIIa were not significantly different between subjects with and without DM (Table 1). PAI-1 did not correlate with plasma TEG-MA (*ρ* = −0.034; *p* = 0.79).

In multivariable linear regression analysis, DM (*p* = 0.003) and fibrinogen (*p* = 0.019) remained significantly associated with plasma TEG-MA after forward stepwise adjustment for clinical variables (Table 4).

## 4. Discussion

The results of our study demonstrate that subjects with diabetes mellitus have increased fibrin clot strength (MA) as measured by TEG in platelet-poor plasma compared to nondiabetics. The association of diabetes mellitus with plasma fibrin clot strength persisted after adjustment for fibrinogen, which influences strength of clot formation measured by TEG. We have recently demonstrated that elevated fibrin clot strength measured by TEG in platelet-poor plasma is associated with increased risk of recurrent myocardial infarction and death after PCI, similar to the findings observed in studies using whole blood TEG assays [17].

In whole blood, maximal clot strength (MA) measured by TEG is the sum of the additive forces generated by aggregating and contracting platelets incorporated into the growing, initially soluble fibrin network, which is cross-linked and stabilized by FXIIIa [13, 20]. In a large cohort study of subjects with coronary artery disease by Lev et al., DM was also independently associated with whole blood (platelet-fibrin) clot strength measured by TEG [21].

In the subgroup of patients in our study who had both whole blood and plasma TEG studies performed, there was a moderate correlation between both measurements for TEG-MA and to a lesser degree for TEG-R and TEG-K. While plasma MA was significantly higher in diabetic subjects, we did not find a significant difference in whole blood MA in our study. This may be due to the smaller number of subjects with whole blood TEG assays completed, as well as possible differences in platelet contribution to clot strength in whole blood TEG between subjects with and without diabetes.

Fibrinogen is an important determinant of fibrin structure and of cross-linked fibrin network strength, and both fibrinogen and factor XIIIa activity influence maximal fibrin clot strength measured by TEG [22]. Fibrinogen has been previously shown to be elevated in subjects with diabetes mellitus [11, 23]. In our study, fibrinogen levels were also higher in subjects with DM, and fibrinogen remained a significant covariate in multivariable linear regression analysis. In contrast, PAI-1 and factor XIIIa were not significantly different between diabetics and nondiabetics.

We did not find any significant correlation between daily insulin dose and plasma TEG-MA among the insulin-dependent subpopulation, while elevated plasma MA was associated with worse glycemic control. Possible mechanisms linking hyperglycemia to a prothrombotic state include effects on gene transcription of coagulation factors, loss of the endothelial layer through glycosylation with a deficiency of nitric oxide, direct glycosylation of coagulation factors altering their activity [24, 25], as well as increased platelet activation [4, 26–29]. Combined hyperinsulinemia and hyperglycemia has been demonstrated to acutely increase circulating tissue factor procoagulant activity (TF-PCA) ninefold in previously healthy subjects [30].

Current recommendations in primary prevention of cardiovascular disease among patients with DM do not yet incorporate use of therapies targeted towards reduction of thrombin formation and fibrin stabilization. However, recent results from the COMPASS study demonstrate a significant reduction in cardiovascular death/myocardial infarction or stroke in patients with stable cardiovascular disease who were treated with rivaroxaban 2.5 mg twice daily in addition to aspirin as compared to patients who were treated with aspirin alone (HR 0.76; 95% CI: 0.66–0.86) [31]. The reduction in risk with rivaroxaban was more pronounced in the diabetic subgroup (HR 0.74; 95% CI: 0.61–0.9) [31]. Future studies will need to assess whether TEG parameters in diabetic patients could be used to risk stratify and develop treatment strategies in diabetic patients with known or suspected cardiovascular disease.

Limitations to our study include that HbA1C values were collected retrospectively and were not standardized to a collection at the time of TEG blood sample collection; as values were only recorded if collected within a three-month window, glycemic control at time of analysis was assumed to be equivalent. We were not able to assess thrombolysis characteristics of patients with DM, as spontaneous thrombolysis generally does not occur with TEG after activation with kaolin and CaCl2.

In conclusion, our data show that diabetes mellitus is associated with increased fibrin clot strength (TEG-MA) as measured in platelet-poor plasma, a marker of thrombotic risk in patients with coronary artery disease. Worse glycemic control as defined by an elevated HbA1C is moderately correlated with higher plasma TEG-MA, whereas there was no statistically significant correlation with use or dosage of insulin. Further studies are warranted to examine the mechanisms associated with increased plasma TEG-MA and to examine the utility of plasma TEG-MA in personalized approaches to guide antithrombotic therapies in patients with diabetes mellitus.

## Figures and Tables

**Table 1:** Baseline variables.

Patient characteristics	Total study population (*n* = 354)	No diabetes mellitus (*n* = 202)	Diabetes mellitus (*n* = 152)	*p* value
Age (years)	57.1 ± 9.9	55.8 ± 9.8	58.8 ± 9.7	0.005
BMI (kg/m^2^)	32 ± 7.3	31.2 ± 7.6	33 ± 6.9	0.018
Male (%)	209/354 (59.3%)	124/202 (61.4%)	85/152 (55.9%)	0.3
African Americans (%)	95/354 (26.8%)	54/202 (26.7%)	41/109 (27%)	0.71
Hypertension (%)	320/354 (90.4%)	172/202 (85.1%)	148/152 (97.4%)	<0.001
Hyperlipidemia (%)	303/354 (85.6%)	160/202 (79.2%)	143/152 (94.1%)	<0.001
Coronary artery disease (%)	310/354 (87.6%)	171/202 (84.6%)	139/152 (91.4%)	0.06
History of myocardial infarction (%)	203/354 (57.3%)	118/202 (58.4%)	85/152 (55.9%)	0.64
History of percutaneous coronary intervention (%)	257/354 (72.6%)	147/202 (72.7%)	110/152 (72.4%)	0.93
History of coronary artery bypass grafting (%)	58/354 (16.4%)	27/202 (13.4%)	31/152 (20.4%)	0.08
Congestive heart failure (%)	63/354 (17.8%)	32/202 (15.8%)	31/152 (20.4%)	0.27
Smoking (%)	162/354 (45.8%)	106/202 (52.5%)	56/152 (36.8%)	0.003
Hemoglobin A1c (%)	n/a	5.82 ± 0.5	7.92 ± 1.8	<0.001
Insulin dependent (%)	n/a	n/a	50/152 (30%)	n/a
Daily insulin dose (units)	n/a	n/a	93 ± 74	n/a
Fibrinogen (mg/ml)	3.7 ± 3.1	3.2 ± 2	4.3 ± 4	0.027
Plasminogen activator inhibitor-1 (ng/ml)	120.8 ± 96	112 ± 89	134.8 ± 106	0.34
Factor XIIIa (%)	91.1 ± 30	92.1 ± 31	89.7 ± 29	0.48

**Table 2:** Thrombelastography measures.

	No diabetes mellitus (*n* = 202)	Diabetes mellitus (*n* = 152)	*p* value
Plasma TEG-R (min)	6.9 ± 3.8	6.6 ± 3.0	0.36
Plasma TEG-K (min)	1.47 ± 1.2	1.38 ± 1.3	0.49
Plasma TEG-MA (mm)	34.1 ± 7.5	37.0 ± 8.1	<0.001
Whole blood TEG-R (min)	5.1 ± 2.2	5.6 ± 1.6	0.26
Whole blood TEG-K (min)	1.5 ± 0.5	1.58 ± 0.5	0.45
Whole blood TEG-MA (mm)	66 ± 5.8	66.6 ± 5.6	0.61

TEG-R = time to fibrin formation; TEG-K = clot formation time; TEG-MA = maximal clot strength. Plasma TEG: *n* = 354. Whole blood TEG: *n* = 74.

**Table 3:** Thrombelastography: insulin-dependent versus non-insulin-dependent diabetes mellitus.

	Insulin-dependent DM (*n* = 50)	Non-insulin-dependent DM (*n* = 102)	*p* value
Plasma TEG-R (min)	6.3 ± 2.3	6.8 ± 3.3	0.37
Plasma TEG-K (min)	1.12 ± 0.6	1.5 ± 1.4	0.08
Plasma TEG-MA (mm)	38.1 ± 9	36.5 ± 8	0.27

TEG-R = time to fibrin formation; TEG-K = clot formation time; TEG-MA = maximal fibrin clot strength.

**Table 4:** Multivariable linear regression analysis.

Variable	*B*	95% confidence interval for *B*	*p* value
Diabetes mellitus	3.5 ± 1.2	1.2–5.8	0.003
Fibrinogen	0.44 ± 0.2	0.07–0.8	0.019

Dependent variable: plasma TEG-MA.
